# Coexistence of aneurysmal subarachnoid hemorrhage and surgically identified pituitary apoplexy: a case report and review of the literature

**DOI:** 10.1186/1752-1947-8-166

**Published:** 2014-05-27

**Authors:** Ren-Xing Song, Dao-Kui Wang, Zhe Wang, Zeng-Wu Wang, Shou-Xian Wang, Guang-Xin Wei, Xin-Gang Li

**Affiliations:** 1 Department of Neurosurgery, Qilu Hospital of Shandong University, 107 Wenhua West Street, Jinan 250012, P.R. China; 2 Department of Neurosurgery, Weifang People’s Affiliated Hospital, Weifang Medical College, 423 Dongfeng West Street, Weifang 261041, China

**Keywords:** Aneurysm, Pituitary apoplexy, Pituitary adenoma, Subarachnoid hemorrhage

## Abstract

**Introduction:**

A ruptured aneurysm associated with a pituitary apoplexy is rare. We present the first case report of the coexistence of a ruptured posterior communicating aneurysm with a surgically discovered pituitary apoplexy where the pituitary apoplexy had not been diagnosed by a pre-operative computerized tomography scan.

**Case presentation:**

A 31-year-old right-handed Chinese woman began to experience severe headache, vomiting and blurred vision which continued for two days. On admission to the hospital, a brain computerized tomography scan demonstrated a small amount of increased signal in the basal cisterns; no evidence of intrasellar and suprasellar lesions was seen. The appearance of her brain suggested aneurysmal subarachnoid hemorrhage. She had nuchal rigidity and reduced vision. There was no extra-ocular palsy and no other neurological deficit. Our patient had no stigmata of Cushing’s syndrome or acromegaly. During an interview for further history, she reported normal menses and denied reduced vision.

Cerebral digital subtraction angiography was subsequently performed, which revealed a 6mm left posterior communicating aneurysm. Urgent left pterional craniotomy was performed. The left ruptured posterior communicating artery aneurysm was completely dissected prior to clipping. At surgery, a suprasellar mass was discovered, the tumor bulging the diaphragma sella and projecting anteriorly under the chiasm raising suspicion of a pituitary tumor. The anterior part of the tumor capsule was opened and a necrotic tumor mixed with dark old blood was removed. The appearance suggested pituitary apoplexy.

Histopathology revealed pituitary adenoma with evidence of hemorrhagic necrosis. Our patient made a good recovery.

**Conclusion:**

Our case report proves that pituitary apoplexy can be coexistent with the rupture of a posterior communicating aneurysm. This association should be considered when evaluating any case of aneurysm. A normal computerized tomography scan does not exclude pituitary apoplexy. Pre-operative magnetic resonance imaging interpretation is required if a pituitary apoplexy is suspected. Craniotomy allows a coexisting aneurysm and pituitary apoplexy to be simultaneously treated.

## Introduction

Pituitary apoplexy is a rare clinical syndrome caused by acute ischemic infarction or hemorrhage of the pituitary gland. The classic presentation of apoplexy includes sudden onset of headache with vomiting, visual disturbance, ophthalmoplegia and altered consciousness. It is classically considered a life-threatening condition, but is commonly misunderstood and often misdiagnosed [1]. It has been largely demonstrated that the incidence of aneurysms associated with pituitary adenoma is significantly higher than for any other brain tumor [2]. A ruptured aneurysm associated with a pituitary apoplexy is rare [3]. We present the first case report of the coexistence of a ruptured posterior communicating aneurysm with a surgically discovered pituitary apoplexy where the pituitary apoplexy had not been diagnosed by a pre-operative CT (computerized tomography) scan.

## Case presentation

**Figure 1:**
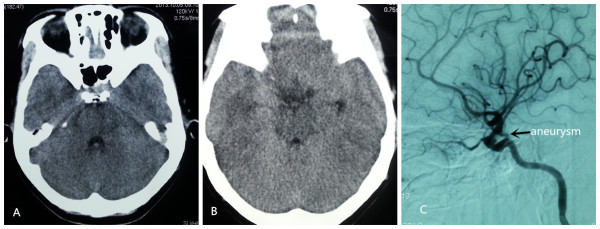
**Pre-operative computed tomography of the brain with cerebral angiogram. (A, B)** A pre-operative brain computed tomography scan demonstrated a small amount of increased signal in the basal cisterns; no evidence of intrasellar and suprasellar lesions was seen. **(C)** Angiogram shows a left posterior communicating artery aneurysm (arrow).

**Figure 2:**
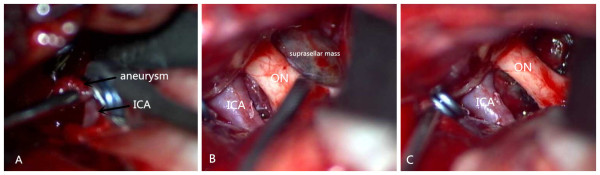
**The aneurysm and suprasellar mass appearance at surgery. (A)** An intraoperative photograph reveals the view after aneurysm clipping. **(B)** This intraoperative photograph reveals a suprasellar mass bulging the diaphragma sella, and projecting anteriorly under the chiasm. ICA = internal carotid artery. ON = optic nerve. **(C)** An intraoperative photograph reveals the view after the tumor was removed. ICA = internal carotid artery. ON = optic nerve.

**Figure 3:**
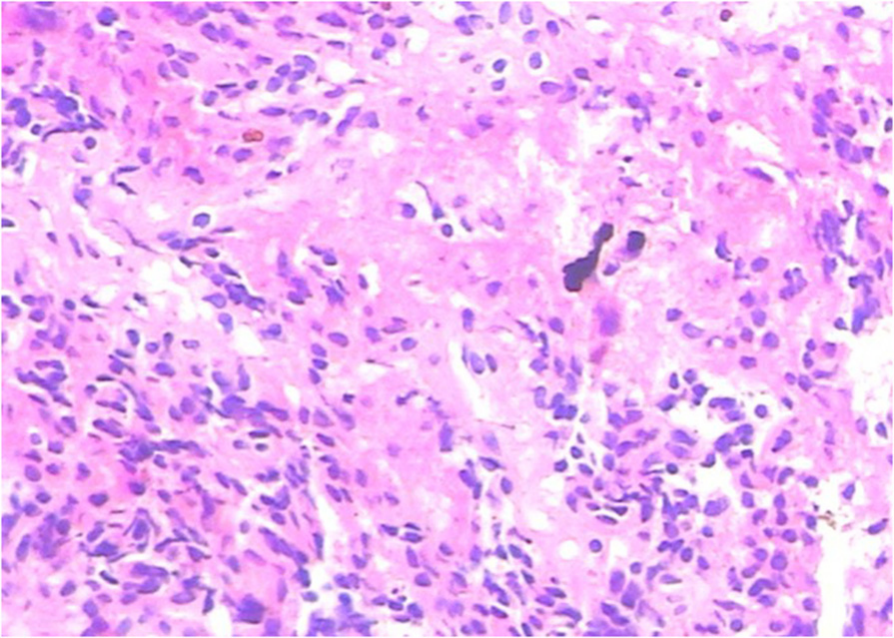
**Histopathology of the surgical specimen.** Area of hemorrhagic necrosis in adenoma (hematoxylin and eosin stain 200×).

**Figure 4:**
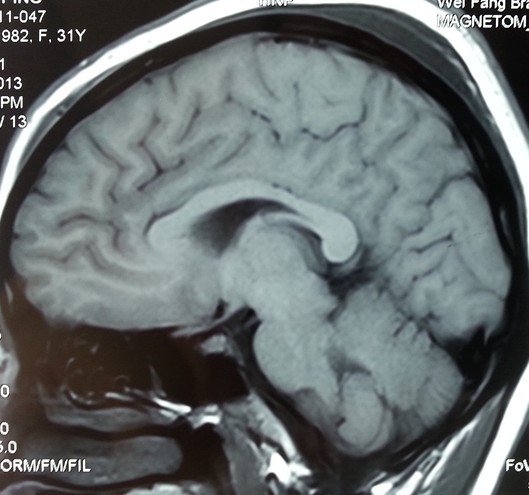
**Postoperative magnetic resonance imaging of the brain.** Postoperative cranial magnetic resonance imaging revealed satisfactory result.

A 31-year-old right-handed Han Chinese woman experienced severe headache, vomiting and blurred vision lasting for two days. On her admission to the hospital, a brain CT scan demonstrated a small amount of increased signal in her basal cisterns, but no evidence of intrasellar and suprasellar lesions were seen (Figure 1A, B). The appearance suggested an aneurysmal subarachnoid hemorrhage.She had nuchal rigidity and reduced vision. There was no extra-ocular palsy, and no other neurological deficit. Our patient had no stigmata of Cushing’s syndrome or acromegaly. During an interview for further history, she reported normal menses and denied reduced vision. A cerebral digital subtraction angiography was subsequently performed, which revealed a 6mm left posterior communicating aneurysm (Figure 1C).Urgent left pterional craniotomy was performed. The left ruptured posterior communicating artery aneurysm was completely dissected prior to clipping. At surgery, a suprasellar mass was discovered, the tumor bulging the diaphragma sella and projecting anteriorly under the chiasm raised suspicion of pituitary tumor (Figure 2B, C). The anterior part of the tumor capsule was opened and a necrotic tumor mixed with dark old blood was removed (Figure 2C). The appearance suggested pituitary apoplexy.Histopathology revealed a pituitary adenoma with evidence of hemorrhagic necrosis (Figure 3).Our patient made a good recovery. Her postoperative vision was fully recovered. Examinations revealed no hormonal or visual disturbances. Postoperative cranial MRI (magnetic resonance imaging) revealed a satisfactory result (Figure 4). She had diabetes insipidus for 10 days postoperatively, which then resolved. She remained asymptomatic and was discharged home on postoperative day 11. She has been followed closely as an outpatient since discharge, and has remained asymptomatic.

## Discussion

The clinical presentation of pituitary apoplexy can vary from patient to patient; some patients may be asymptomatic whereas others may present with features of a subarachnoid hemorrhage [4]. Our patient presented with features of aneurysmal subarachnoid hemorrhage. With her case, we prove that if an aneurysm is present, the possibility of it coexisting with a pituitary adenoma should be considered. Diagnosis of a pituitary adenoma was frustrated by the normal CT appearance in our patient’s case. An MRI is superior to CT for the diagnosis of pituitary region tumors. Routine MRI should provide the pre-operative assessment to rule out a coexisting pituitary adenoma.

In previous cases, the vast majority of coexisting pituitary adenomas were diagnosed before surgery; most aneurysms were unruptured aneurysms and located close to the pituitary tumor [5,6]. Laidlaw *et al*. [7] reported a case of posterior communicating aneurysmal subarachnoid hemorrhage with coexisting pituitary apoplexy. However, unlike our patient, in their patient, the pituitary tumor was revealed by CT scan before surgery. The case we present here is the coexistence of an aneurysmal subarachnoid hemorrhage and a surgically discovered pituitary apoplexy which had not been identified on CT before surgery. To our knowledge, this is the first such report.

The pathogenesis of the pituitary apoplexy is poorly understood. It is uncertain whether pituitary apoplexy was the result of the aneurysm or whether pituitary apoplexy resulted in the aneurysm. Some reports illustrated that pituitary apoplexy mimics an aneurysm [5,8]. Hypotheses for this phenomenon have been proposed, which include local circulatory mechanisms, growth hormone production that induces artery dilation, a direct mechanical effect resulting from the pituitary apoplexy on the vasculature, and/or direct infiltration by the tumor, predisposing to aneurysm formation [9,10].

On the other hand, rupture of an intracranial aneurysm may simulate pituitary apoplexy. Different mechanisms have been proposed and include acute dilatation of the artery aneurysm causing acute compression of the residual pituitary gland and inflicting direct tissue damage to the pituitary gland or stalk, or ischemic changes attributable to the raised intracranial pressure, and/or because of the later effects of vasospasm [3,11,12].

However, a clear mechanism of coexistence of two pathologies has not been found. In the present case, the development of the pituitary apoplexy is not clear, but we hypothesize that due to the raised intracranial pressure at the time of the subarachnoid hemorrhage, a compromised superior hypophyseal arterial and/or meningohypophyseal trunk may result in ischemia and the resultant apoplexy.

## Conclusion

Our case proves that a pituitary apoplexy can be coexistent with the rupture of a posterior communicating aneurysm; this association should be considered when evaluating any case of aneurysm. A normal CT scan does not exclude pituitary apoplexy. Pre-operative MRI interpretation is required if a pituitary apoplexy is suspected. Craniotomy allows a coexisting aneurysm and a pituitary apoplexy to be simultaneously treated.

## Consent

Written informed consent was obtained from the patient for publication of this case report and any accompanying images. A copy of the written consent is available for review by the Editor-in-Chief of this journal and will be provided on request.

## Abbreviations

CT: Computerized tomography; ICA: Internal carotid artery; MRI: Magnetic resonance imaging; ON: Optic nerve.

## Competing interests

The authors declare that they have no competing interests.

## Authors’ contributions

R-XS, D-KW and Z-WW were involved in the initial writing of the manuscript. R-XS provided major editing changes. ZW, S-XW and G-XW were primarily involved in the care of our patient. X-GL provided intellectual contributions to the content of the manuscript as well as editorial assistance. All authors have read and approved the final version of the manuscript.
